# Testicular Compartment Syndrome Post Bilateral Microdissection Testicular Sperm Extraction: A Case Report and Review of the Current Literature

**DOI:** 10.1155/criu/2710616

**Published:** 2026-04-15

**Authors:** Catherine Larard, Katie McComb, Ivo Donkov, Alberto Coscione, Panagiotis Nikolinakos, Karl H. Pang

**Affiliations:** ^1^ Faculty of Medicine, Imperial College London, London, UK, imperial.ac.uk; ^2^ Department of Urology, West Middlesex University Hospital, Chelsea and Westminster Hospital NHS Foundation Trust, London, UK, nhs.uk; ^3^ Department of Urology, Chelsea and Westminster Hospital, Chelsea and Westminster Hospital NHS Foundation Trust, London, UK, nhs.uk; ^4^ Department of Pediatric Surgery, National and Kapodistrian University of Athens, Athens, Greece, uoa.gr; ^5^ Division of Surgery and Interventional Science, University College London, London, UK, ucl.ac.uk

**Keywords:** andrology, mTESE, surgery, testicular compartment syndrome, urology

## Abstract

**Background:**

Testicular compartment syndrome (TCS) is a rare phenomenon with around 14 cases reported in the literature. It occurs when extraluminal compression or increased venous resistance leads to raised intratesticular pressure. The fibrous tunica albuginea covering the testes does not distend, meaning the pressure is not relieved and compartment syndrome can develop. The ensuing reduction in perfusion and subsequent reperfusion injury can compromise testicular viability, making prompt identification and early surgical intervention essential in optimising outcomes.

**Case Presentation:**

A 41‐year‐old male presented to the emergency department with acute severe bilateral testicular pain 1 day after bilateral microdissection testicular sperm extraction (mTESE) for primary infertility and azoospermia. He reported a gradual onset of bilateral testicular pain throughout the day accompanied by oozing and bleeding from the surgical wound. The pain escalated with time, and both testicles were exquisitely tender on palpation. Ultrasonography (US) demonstrated diffuse scrotal sac thickening, heterogenous testicles with hyperaemia on Doppler flow and bilateral hyperaemic enlargement of both epididymi. Thus, an emergency scrotal exploration was performed with evacuation of a left intratesticular haematoma and a right peritesticular haematoma. This restored perfusion to both testes and the patient had an uneventful postoperative course. A follow‐up US after 6 weeks revealed both testicles had a reduction in volume and reduced blood flow.

**Conclusion:**

TCS is a previously undocumented complication of mTESE that should be considered in any patient presenting with pain out of proportion to clinical findings. Although the diagnosis is primarily clinical, US features, such as initial hyperaemia progressing to reduced blood flow, can support the diagnosis. Early surgical intervention to decompress the testis by incising the tunica layers is critical to reduce intratesticular pressure, restore perfusion and preserve testicular viability.

## 1. Introduction

Compartment syndrome is a surgical emergency where pressure within a closed fascial compartment increases to levels that compromise blood flow, resulting in a potentially irreversible tissue ischaemia. It is most common within the anterior compartment of the leg but can very rarely occur within the testes.

In cases of extraluminal compression or increased venous resistance, the fibrous tunica albuginea covering the testes does not distend leading to the development of compartment syndrome. Testicular compartment syndrome (TCS) is defined by decreased perfusion of microcirculation within testicular tissue secondary to compression. Damage to testicular tissue is further compounded by inflammation following reperfusion termed reperfusion injury. TCS is a rare but severe phenomenon with 14 cases reported in the literature, of which two cases progressed to the loss of testis viability and subsequent orchidectomy.

Here, we present the case of a 41‐year‐old with bilateral TCS after undergoing a microdissection testicular sperm extraction (mTESE) for primary infertility and azoospermia. It highlights the need to remain vigilant for postoperative complications even in relatively minor procedures and adds to the literature regarding the safety profile of mTESE.

## 2. Case Report

### 2.1. Clinical Presentation

A 41‐year‐old male presented to the emergency department with increasing scrotal pain and swelling following a bilateral mTESE procedure the day before. The patient suffered from primary infertility and azoospermia. He had previously undergone a successful right‐sided mTESE abroad, but in vitro fertilisation (IVF) was unsuccessful. His past medical history was otherwise unremarkable. Prior investigations, including genetic testing (karyotype, cystic fibrosis and Y‐deletion), early morning fasted testosterone was 15.5 nmol/L, and testicular examination was within normal limits. Follicle‐stimulating hormone (FSH) was high at 19.6 IU/L. Preoperative ultrasonography (US) revealed testes that were normal in size with no masses, varicoceles or hydroceles.

### 2.2. Diagnostic Approach

The differential diagnoses focused on vascular versus infectious causes for his testicular pain and swelling. On examination, the wound was clean and there was mild testicular swelling with significant pain on palpation. The main abnormality to note on laboratory tests was a mildly elevated C‐reactive protein (CRP) of 7.8 mg/L. Whereas a full blood count and wound swabs were unremarkable, making infection less likely. Scrotal US demonstrated no focal testicular lesions or fluid collections, but revealed marked hyperaemia of both testes and the scrotum. In view of the patient′s persistent pain and concern for vascular compromise, he was taken back to theatre for an emergency scrotal exploration.

### 2.3. Management and Outcome

Operative findings revealed a tense left testis with an engorged left epididymis upon scrotal exploration. The left tunica albuginea was opened, revealing a small intratesticular haematoma; however, no active bleeding was observed. The haematoma was evacuated with immediate improvement in epididymal swelling indicating a relief in pressure. The left tunica albuginea was then closed.

On the right side, a peritesticular haematoma was identified and evacuated. The right testis did not look tense or swollen, hence not explored.

**Figure 1:**
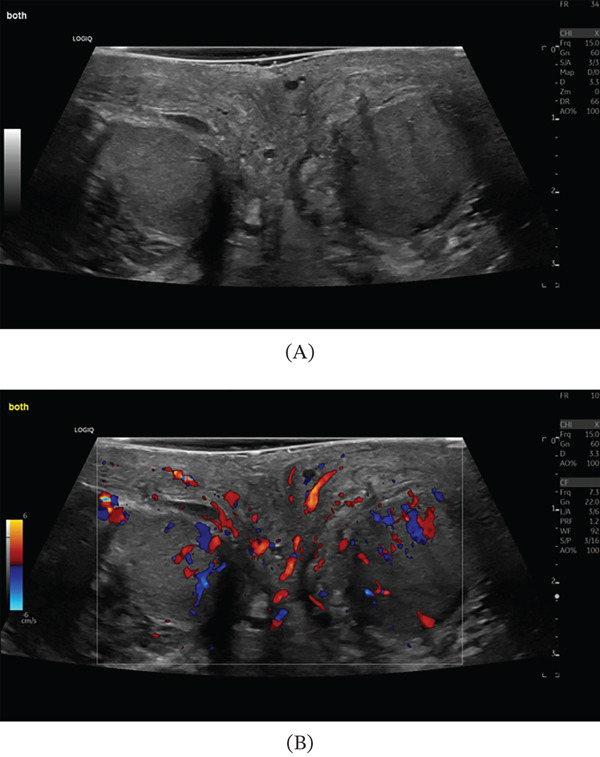
Immediate postoperative ultrasound (US) of both testes; (A) conventional US of both testes and (B) Doppler US of both testes demonstrating hyperaemia.

**Figure 2:**
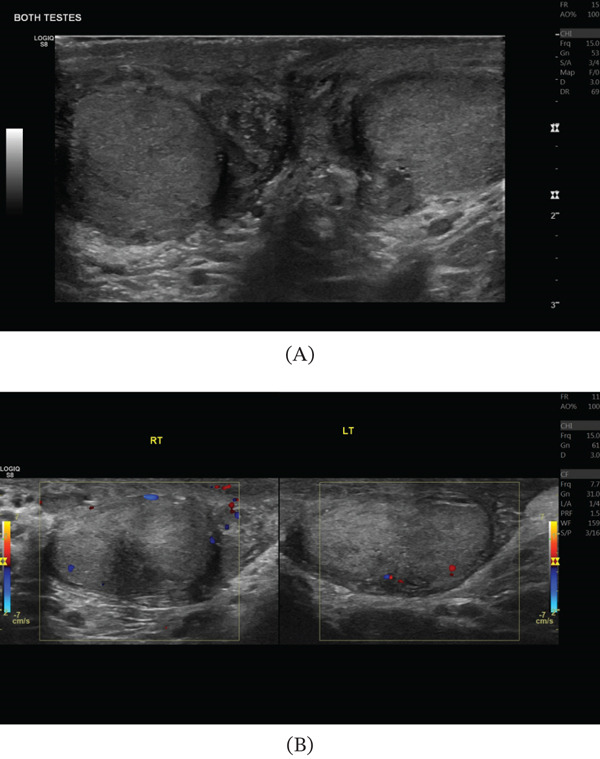
Six‐week postoperative ultrasound (US) of both testes; (A) conventional US of both testes and (B) Doppler US of both testes demonstrating reduced blood flow especially on the right and relative reduction in testicular volume to 3 cc bilaterally.

The patient′s postoperative course was uncomplicated. He remained in hospital for a total of 3 days afterwards for pain management, intravenous antibiotics and regular US monitoring. His blood tests were largely normal and demonstrated a down trending CRP, which was 9.6 mg/L on discharge. Postoperative Day 6 US examination revealed reduced blood flow to both testes, more on the right (Figure 1). Nevertheless, the patient was counselled on the risk of subsequent testicular atrophy and scheduled for a follow‐up US 6 weeks after discharge. At 6 weeks, a subsequent US revealed that both the right and left testes had reduced in volume to 3 cc and demonstrated reduced blood flow bilaterally in colour Doppler (Figure 2). The patient has been booked for andrology review and will have their testosterone monitored in a primary care setting.

The mTESE procedure was successful and obtained one vial of twitching sperm and four vials of immotile sperm. Histopathological analysis demonstrated a Sertoli cell–only pattern without evidence of germ cell neoplasia in situ. [1] Despite the successful sperm retrieval, the outcome of IVF is not yet known.

## 3. Discussion

mTESE is considered the gold standard surgical technique for retrieving sperm in men with nonobstructive azoospermia [1]. Unlike conventional TESE, which involves random sampling of testicular tissue, mTESE uses an operating microscope to identify larger, more opaque seminiferous tubules—those most likely to contain sperm. These tubules are selectively biopsied, and the tissue is immediately examined for spermatozoa. The use of microscopy enables a more targeted and efficient retrieval, increasing success rates while minimising unnecessary tissue damage [2].

In this case, one vial of twitching sperm and four vials of immotile sperm were obtained, which is positive given that studies suggest 55% of mTESE procedures do not collect any sperm [3]. The main factor influencing whether sperm collection is successful is the histopathological analysis, which usually demonstrates one of three cell type patterns—hypospermatogenesis, cell maturation arrest or Sertoli cells–only pattern in those with nonobstructive azoospermia [4]. Of those, a Sertoli cell–only pattern is associated with the worst rates of sperm retrieval, due to the lack of germ cells needed for spermatogenesis [5, 6]. This patient had a Sertoli cell–only pattern, obtained from random biopsies taken from the upper and lower pole of the testes. Motile sperm was found; therefore, the patient had heterogeneous testes, and the site of motile sperm found was likely not from the poles where the biopsies were taken.

Previous literature does demonstrate that sperm collection can still be successful with this histological pattern, again, likely associated with heterogenous testes [4, 7]. The patient′s FSH was high, which also reflects hypospermatogenesis. Studies suggest that high FSH correlates with less chance of a successful mTESE procedure, whereas others suggest that high levels do not significantly change the likelihood of a positive outcome [8–10]. For redo mTESEs, systematic mapping biopsies could be taken to identify areas of spermatogenesis for subsequent targeted retrieval.

This case report highlights TCS as a complication of mTESE surgery—a rare but potentially serious condition.

Current patient information materials typically list bruising and postoperative pain as the most common complications, with bleeding or infection requiring further intervention occurring in approximately 1 in 50 cases [11, 12]. However, it is worth considering that in some of these cases with bleeding, TCS may have been prevented by timely surgical management. Prompt recognition and intervention are therefore essential to reducing morbidity from this rare complication. Notably, a specialised centre in Italy recommends a routine assessment of both testes for haematoma formation prior to discharge, possibly to prevent missed cases of evolving TCS [13]. Routine pretesting of this patient, including hormone profile and US, did not identify any specific predisposing factors. Furthermore, there was no family history of conditions that might contribute to TCS such as thrombophilia. The absence of predisposing risk factors highlights the need to keep TCS as a differential in all cases. A more detailed examination of the available literature suggests that, despite the absence of clearly defined risk groups, there may be shared mechanistic features across cases. Across reported cases, TCS consistently occurs following a precipitating insult that either increases intratesticular volume (e.g., haemorrhage, oedema and hydrocele) or impairs venous outflow (e.g., torsion, infection and trauma). Despite differing aetiologies, these cases appear to converge on a common pathophysiological pathway involving rising intratesticular pressure within a nondistensible tunica albuginea, resulting in microvascular compromise, ischaemia and reperfusion injury.

Some degree of Leydig cell damage is inevitable given their location adjacent to seminiferous tubules, but as many as 1 in 50 patients experience testicular atrophy and require testosterone replacement due to postoperative ischaemia [11, 12]. In this case, a testicular US performed at 6 weeks demonstrated reduced blood flow and testicular atrophy. Moving forward, testosterone levels will be monitored, and the patient is due an andrology follow‐up.

We conducted a review of the literature regarding TCS and the results are summarised below (Table 1). The PubMed, Ovid Embase and Cochrane databases were searched using the following subject headings: ([testes or testis or testic∗] AND [compartment syndrome]). This generated 190 articles, which were then screened for relevance by an investigator (C.L.). After removing articles that were irrelevant, duplicates or preclinical studies, there were 10 articles containing the 14 cases below. This highlights the heterogeneity in cause, US findings and outcomes among cases (Table 1).

**Table 1:** Similar case reports of testicular compartment syndrome.

Study	Age (years)	Cause	US findings	Time to intervention	Outcome
Johnson‐Smith et al. [14]	40	Epididymitis with hydrocele	Hypertrophic, hyperaemic and heterogenous epididymis with REDF	Urgent	Tunica albuginea dissected and testis fully reperfused
Johnson‐Smith et al. [14]	37	Epididymitis	Hypervascular, enlarged left epididymal tail with REDF	Around 48 h	Orchidectomy
Phan et al. [15]	11	Trauma	Enlarged and heterogenous testis with diminished blood flow	Around 24 h	Tunica albuginea dissected, tunica vaginalis flaps made and testis fully reperfused
Drone et al. [16]	28	Varicocele in patient with HIV	Enlarged, hypervascular testes	Multiple interventions over 9 days	Bilateral orchidectomy secondary to infection
Hanna et al. [17]	20	Trauma	Normal echotexture and vascularity of left testis; hypoechoic texture of right testis with no blood flow	Within 4 h	Fasciotomy and successful reperfusion of left testis right orchidectomy
Kutikov et al. [18]	14	Testicular torsion	No blood flow to testis	Within 7 h	Tunica albuginea dissected, tunica vaginalis flap made and testis reperfused
Kutikov et al. [18]	16	Testicular torsion	No blood flow to testis	Within 6 h	Tunica albuginea dissected, tunica vaginalis flap made and testis reperfused with small area of infarction
Kutikov et al. [18]	11	Testicular torsion	N/A	Within 6 h	Tunica albuginea dissected, tunica vaginalis flap made and testis reperfused
Douglas et al. [19]	55	Hydrocele with trauma	No blood flow to testis	Urgent	Aspiration of hemi scrotum, testicle reperfused
Chen et al. [20]	60	Hydrocele	Absent and intermittent REDF	Urgent	Aspiration with subsequent hydrocelectomy
Suzuki et al. [21]	14	Testicular torsion	No blood flow	9 h	Detorsion, tunica albuginea incised
Kikkawa et al. [22]	13	Testicular torsion	Results not available	6 h	Detorsion, tunica albuginea incised, covered with tunica vaginalis flap
Kikkawa et al. [22]	16	Testicular torsion	Results not available	10 h	Detorsion, tunica albuginea incised and covered with tunica vaginalis flap
Sharifi et al. [23]	22	No precipitant	Not conducted	3 h after presenting to hospital	Tunica albuginea incised, testis reperfused

Abbreviations: HIV, human immunodeficiency virus; REDF, reversed end‐diastolic flow; US, ultrasonography.

This case of TCS arose as a direct complication of mTESE; all prior cases arose from external mechanical insults, infection, torsion or fluid accumulation. Furthermore, the bilateral involvement in the present case is exceptional—the majority of published TCS cases are unilateral. Ultrasonographically, the initial finding of bilateral hyperaemia with preserved Doppler signal, rather than absent or reversed end‐diastolic flow, distinguishes this case from the majority of prior reports and represents an early‐stage finding of important diagnostic teaching value: hyperaemia should prompt heightened vigilance for evolving TCS rather than false reassurance, particularly in the first 24–48 h following scrotal surgery. The time to intervention was urgent in the present case, consistent with the favourable outcomes seen across the literature; the two cases requiring orchidectomy were characterised by delayed presentation or prolonged ischaemia.

There is potential inaccuracy in making a diagnosis of TCS due to a lack of standardisation regarding the investigations. The gold‐standard investigation of intracompartmental pressure measurements is rarely used due to practical limitations regarding the need for emergency reperfusion, the potential for further damage and the lack of standardised guidelines required for interpretation [18, 24]. This patient demonstrated an elevated CRP, but this is by no means a specific or sensitive result to aid in the diagnosis. Clinical examination supported by US remains the most accessible diagnostic pathway; however, it lacks specificity and often identifies only late signs, such as absent or reduced testicular blood flow [12]. Prior to the development of reduced perfusion in TCS, hyperaemia may be detected on US, as observed in this case. However, hyperaemia is also commonly seen in testicular torsion, meaning that US cannot reliably differentiate between these clinically similar conditions [25]. As a result, many diagnoses rely primarily on clinical suspicion rather than confirmatory imaging or intracompartmental pressure measurements, increasing the risk of missed or misdiagnosed cases [26].

Despite the diagnostic challenges, prompt management generally leads to favourable outcomes. Of the nine published case reports, only two required an orchidectomy, even in cases where blood flow was absent on the preoperative US. Intraoperative management creating a flap from the tunica vaginalis to extend the tunica albuginea appears to be an effective strategy in cases where directly suturing the tunica albuginea leads to a recurrence of raised pressures [15]. In addition, reperfusion injury can worsen or outlast the damage suffered to testicular tissue from the compartment syndrome. Reactive oxygen species are formed following the ischaemic period, which are particularly damaging to Leydig cells given the high rate of metabolism, potentially exacerbating male subfertility in an otherwise viable testicle [26]. Preclinical studies have suggested administering hirudin, an antioxidant with anti‐inflammatory properties, does reduce cell death from oxidative stress, offering a potential avenue to improve the long‐term management of compartment syndrome [27, 28].

## 4. Conclusion

TCS is a rare but important diagnosis that should be considered in any patient presenting with significant testicular pain, particularly when the severity is out of proportion to clinical findings. This case highlights TCS as a serious complication arising from a relatively minor procedure, mTESE. Although the diagnosis remains primarily clinical, US findings—such as initial hyperaemia followed by diminished blood flow together with scrotal pain and swelling—may aid in identification. Prompt surgical intervention to decompress the testis by incising the tunica albuginea is essential to reduce intratesticular pressure, restore perfusion and preserve testicular viability. Further research into optimal surgical procedures and postoperative management may help minimise cellular damage and improve outcomes in otherwise salvageable testes.

## Author Contributions

Panagiotis Nikolinakos and Karl H. Pang are joint senior authors.

## Funding

No funding was received for this manuscript.

## Conflicts of Interest

The authors declare no conflicts of interest.

## Data Availability

Data sharing is not applicable to this article as no datasets were generated or analysed during the current study.
